# An observational study demonstrates human-adapted *Staphylococcus aureus* strains have a higher frequency of antibiotic resistance compared to cattle-adapted strains isolated from dairy farms making farmstead cheese

**DOI:** 10.1186/s12917-024-03910-6

**Published:** 2024-02-27

**Authors:** Ashma Chakrawarti, Christine L. Casey, Ariela Burk, Robert Mugabi, Amanda Ochoa, John W. Barlow

**Affiliations:** 1 Department of Animal and Veterinary Sciences, University of Vermont, Burlington, VT USA; 2 Kentucky Department of Fish and Wildlife Resources, Frankfort, KY USA; 3 Department of Pathobiological Sciences, University of Wisconsin-Madison, Madison, WI USA; 4 Department of Veterinary Diagnostic & Production Animal Medicine, Iowa State University, Ames, IA USA; 5 King Arthur Baking Company, Norwich, VT USA

**Keywords:** *Staphylococcus aureus*, Antimicrobial resistance, Strain typing, Mastitis, Spillover, Dairy Farm, Farmstead cheese

## Abstract

**Background:**

*Staphylococcus aureus* is a multi-host zoonotic pathogen causing human and livestock diseases. Dairy farms that make artisan cheese have distinctive concerns for *S. aureus* control. Antimicrobial-resistant (AMR) *S. aureus* is a public and animal health concern. There is a need to study the population structure of AMR *S. aureus* at the human-animal interface and understand the path of zoonotic transmission. This cross-sectional observational study aimed to assess the genetic diversity and AMR patterns of *S. aureus* isolated from cattle and humans on conventional and organic Vermont dairy farms that produce and sell farmstead cheese.

**Results:**

A convenience sample of 19 dairy farms in Vermont was enrolled, and 160 *S. aureus* isolates were collected from cow quarter milk (CQM), bulk tank milk (BTM), human-hand and -nasal swabs. After deduplication, 89 isolates were used for the analysis. Sequence types (STs) were determined by multilocus sequence typing and cataloged to the PubMLST database. Nine defined and five novel STs were identified. For BTM and CQM samples, six STs were identified within cow-adapted CC97 and CC151. Two human-adapted STs were isolated from BTM and CQM. Seven human-adapted clonal complexes with eight STs were identified from human samples. One cow-adapted ST was isolated from a human. Antimicrobial susceptibility of the isolates was tested using disc diffusion and broth microdilution methods. Approximately 27% of the isolates were beta-lactam resistant and *blaZ* gene-positive. *S. aureus* isolates from human swabs were more likely to carry *blaZ* compared to isolates from CQM or BTM. *S. aureus* isolated from cows and humans on the same farm belonged to different STs.

**Conclusion:**

Humans were more likely to carry beta-lactam-resistant *S. aureus* compared to cows, and on organic farms only human-adapted *blaZ* positive STs were isolated from BTM. Moreover, we identified potential spillover events of *S. aureus* sequence types between host species. The presence of penicillin-resistant-human-adapted *S. aureus* on both organic and conventional dairy farms highlights a “One Health” concern at the junction of public and animal health requiring further surveillance.

**Supplementary Information:**

The online version contains supplementary material available at 10.1186/s12917-024-03910-6.

## Background

*Staphylococcus aureus* is a major cause of bovine intramammary infection on dairy farms. Infected mammary glands are the primary source of contagious *S. aureus* mastitis transmission among quarters and cows during milking. Other body sites and housing environments can also act as reservoirs for *S. aureus* and may be associated with sporadic or incidental intramammary infections [1, 2]. Farm workers are another potential source of *S. aureus* on dairy farms [3–6]. *S. aureus* is commonly present in the anterior nares of approximately 30% of the human population and causes food poisoning, skin infections, bacteremia, endocarditis, and other diseases in humans [7–9]. *S. aureus* is also a “Priority 2 pathogen” on the World Health Organization's (WHO) list of global antibiotic-resistant bacteria [10]. Staphylococcal infections are defined as amphixenoses, infections transmitted in both directions, i.e., from animals to humans and vice versa [11]. Other terms have been used to describe the directionality of transmission (i.e., anthropozoonoses and zooanthroponoses), however, the WHO Joint WHO/FAO expert committee on zoonoses recommended “zoonoses” are “diseases and infections which are naturally transmitted between vertebrate animals and man” [12].

Globally, most antimicrobial use on dairy farms is attributed to mastitis control [13]. An exception is US organic dairy farms where antimicrobial use is prohibited, and animals receiving antibiotic treatments must be permanently removed from organic production [13]. The usage of antibiotics creates selective pressure, driving antimicrobial resistance (AMR) in both human and veterinary medicine [14, 15]. The spread of AMR *S. aureus* strains between livestock and humans underscores the need for a One Health approach, with several studies indicating the zoonotic potential of certain *S. aureus* lineages [4, 16, 17]. Since cattle domestication, the proximity of humans and cows or the consumption of raw milk and dairy products have increased the risk of *S. aureus* spillovers [16, 18–20]. This necessitates continued monitoring of the genetic diversity of *S. aureus* at the human-animal interface to inform potential spillover events. Multilocus sequence typing (MLST) has greatly advanced our understanding of the population structure of *S. aureus* strains and their epidemiology and host specificity [5, 21–23].

Artisan or artisanal cheese is produced in part by hand, in small batches, using traditional methods. Farmstead cheeses are made on the same farm where the animals that supply the milk are raised and milked [24]. Farmstead cheese production systems may be especially unique because the farm workers may engage in all segments of the dairy production chain, from animal management and milking to cheese production. Farmstead cheese producers may be a source of *S. aureus* milk contamination during harvest or cheese production [24, 25].

Despite efforts to address the issue of AMR, there is limited surveillance data on *S. aureus* strain diversity and AMR profiles of isolates from farm workers and cattle on dairy farms that produce and sell farmstead or artisan cheese in the United States [18]. This lack of information hampers the ability to effectively implement the One Health approach to prevent the spread of AMR pathogens [26]. Understanding the epidemiology, ecology, and antibiotic resistance of *S. aureus* is crucial to improving our knowledge of the factors driving the selection, maintenance, and spread of AMR pathogens in farm systems. To address this gap, our study aimed to assess the strain diversity and antimicrobial susceptibility of *S. aureus* isolated from humans and cows on dairy farms producing and selling farmstead or artisan cheese. We were especially interested in antimicrobial sensitivity to common antibiotics used in veterinary and human medicine and evaluating the relationship between epidemiological predictors (such as isolate host source, strain type, and farm type) and AMR phenotypes and genotypes among *S. aureus* isolates on these farms. An exploratory objective was to identify potential evidence of *S. aureus* spillover events occurring on the enrolled dairy farms.

## Results

### Descriptive Analysis

This study included 41 human participants (1 to 4 per farm) from 19 farms, bulk tank milk (BTM) samples from all 19 farms, 589 cows (3 to 204 per herd) from 17 of the participating farms, and 13 dogs from 9 of the farms. The distribution of isolates collected by source is summarized in Table 1. *S. aureus* was isolated from 15 (36.6%) humans from either hand (*n* = 8) or nasal (*n* = 13) swabs on 13 farms. *S. aureus* was found in 44 quarters of 35 cows on 11 farms. The frequency of *S. aureus* in BTM was 63.1% (12/19 farms). At the herd level, for the 17 farms where we have samples from all three sources, *S. aureus* was isolated from the bulk tank milk on 11 farms, from one or more human samples on 10 farms, and from one or more individual cows on 11 farms. When the individual human and cow level samples are clustered at the farm level, the frequency of positive farms did not differ by source; *S. aureus* was isolated from humans on 13/19 (68%) of farms and from individual cows on 11/17 (65%) of farms (Additional file 1). *S. aureus* was rarely isolated from the dog-nasal swabs, with only 2 isolates identified as *S. aureus.* One dog isolate was lost to follow-up. In summary, 162 isolates were confirmed to be *S. aureus* (i.e., gram-positive, catalase-positive, coagulase-positive, *nuc*-PCR-positive cocci). Overall, 94.73% (18/19) of herds were *S. aureus *positive from at least one of the three sources (Human, BTM, or CQM), and five herds were positive from all three sources. The single available dog isolate was not included in our analysis, leaving 160 isolates for further analysis. Because multiple isolates of the same strain type were collected from individual samples, deduplication of the 160 isolates was performed to avoid over-representation of strain diversity and provide unbiased antimicrobial resistance prevalence estimates. reducing the total number of isolates to 89. The list of the 89 isolates, their metadata, and the number of duplicates per isolate is included in additional file 1. Of these, 79.7% (71/89) of *S. aureus* isolates were collected from 15 conventional farms and 20.2% (18/89) from 4 organic dairy farms.

**Table 1:** Summary of collection, isolation, and identification of *S. aureus* across different sample sources on 19 farmstead cheese producer farms in Vermont

Source	No. of farms sampled	No. of participants/ samples	Total isolates examined	Gram-positive, catalase-positive cocci isolated	Coagulase positive and *nuc*-positive isolates	Number of farms where *S. aureus* was isolated from this source	Notes
Human	19	41 humans (1 to 4 participants per farm)	717 (352 isolates from hand swabs and 365 isolates from nasal swabs)	580 (290 isolates from hand swabs and 290 isolates from nasal swabs)	69 (12 isolates from hand swabs and 57 isolates from nasal swabs	13	*S. aureus* isolated from 15 humans
BTM	19	19 samples (1 per farm)	211	187	36	12	*S. aureus* isolated from 12 BTM samples
CQM	17	589 cows sampled (3–204 cows per herd)	584	395	55	11	*S. aureus* found in 44 quarters of 35 cows from 11 farms
Dogs	9	13	65	52	2	2	1 isolate lost to follow-up and the available one was not included in the analysis
Total			1577	1214	162		The final total was 160 after removing dog isolates

### MLST Profiles

**Fig. 1:**
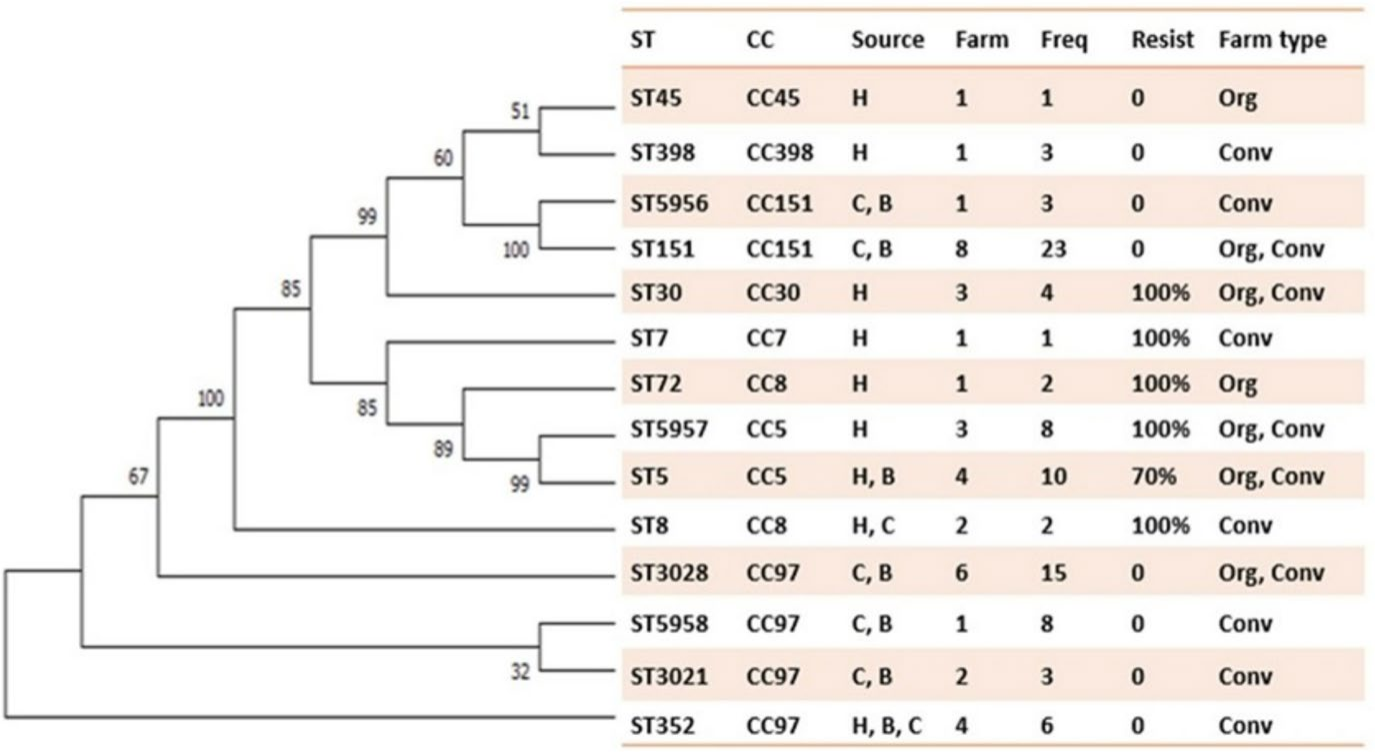
Phylogenetic relationship of *S. aureus* MLST profiles. Dendrogram based on sequence variation in 7 core genes of the *S. aureus* MLST scheme, showing the frequency and number of farms where a strain type was isolated and the association between specific STs and beta-lactam antibiotic resistance. Freq: Frequency; CC: Clonal Complex; Resist: percentage of isolates for the respective strain types resistant to ampicillin/penicillin and positive on *blaZ* PCR; Source: Sample source from which the strains were isolated; H: Human Nose/Hand. B: Bulk Tank Milk, C: Cow quarter milk; Farm type: Org: Organic Farm, Conv: Conventional Farm

**Fig. 2:**
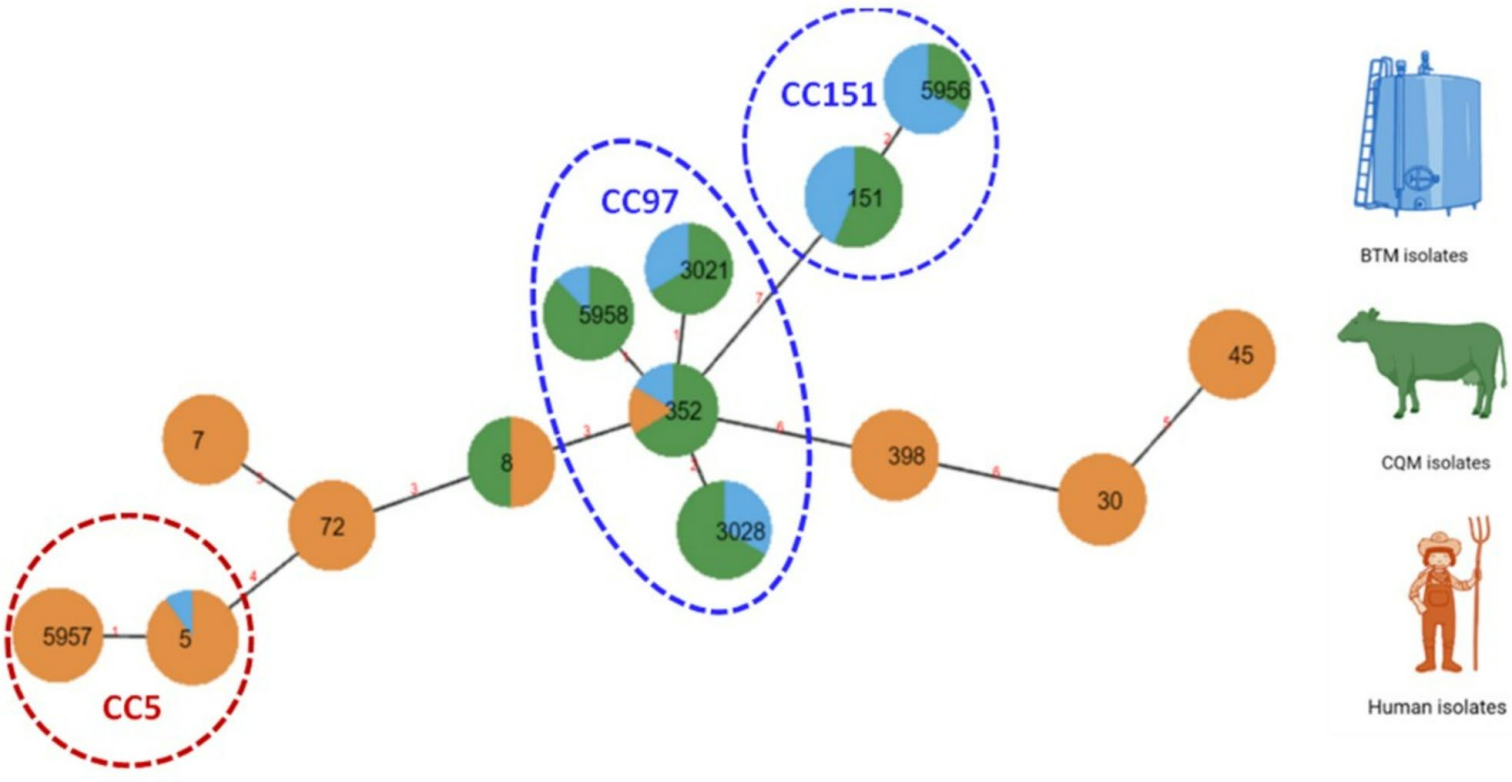
Minimum spanning tree of *S. aureus* isolates. Eighty-nine deduplicated *S. aureus* isolates from BTM (bulk tank milk), CQM (cow quarter milk) and farm workers based on MLST profiles. Each node represents a sequence type (ST) with the size of diameter representing the number of isolates belonging to that ST. The color represents the source of the isolates. The number on the lines shows the number of allelic differences between ST nodes

The 89 deduplicated *S. aureus* isolates were classified into 14 different MLST sequence types (STs) and eight clonal complexes (CCs) (Fig. 1). Of these, 9 were known STs (ST5, ST7, ST8, ST30, ST45, ST72, ST151, ST352, and ST398) and 5 were novel STs (ST3021, ST3028, ST5956, ST5957, and ST5958). The distribution of STs and CCs varied among different sources and farms, with multiple STs and CCs present on some farms. The dominant STs were ST151 (*n* = 23), ST3028 (*n* = 15), ST5 (*n* = 10), and ST5958 (*n* = 8). The strains isolated from the organic farms were CC5, CC8, CC30, CC45, CC97, and CC151, and CC5, CC7, CC8, CC30, CC97, CC151, and CC398 were isolated from the conventional farms (Fig. 2).

Five STs (ST3021, ST151, ST5956, ST3028, and ST5958) belonging to CC97 and CC151 were isolated from bovine sources (BTM and CQM). Six STs (ST45, ST30, ST72, ST5957, ST7, and ST398) were isolated from human sources. These STs clustered within clonal complexes CC45, CC30, CC7, CC5, CC8, and CC398. The remaining STs (ST5, ST8, and ST352) were isolated from bovine and human sources on these farms (Fig. 2). The single dog isolate available was ST45, and this strain type was not identified from any other source on the farm (farm 2) where this dog lived. However, ST45 was isolated from a human-nasal swab sample from a different farm (farm 8).

### Antibiotic Susceptibility Tests

Isolates were resistant to sulfadimethoxine (67%, 60/89), beta-lactams (27%, 24/89), erythromycin (9%, 8/89), lincomycin (2%, 2/89), tetracycline (1%, 1/89), and pirlimycin (1%, 1/89). Twenty of 30 (67%) human isolates were resistant to two or more antibiotic classes. These isolates were resistant to penicillin and sulfadimethoxine (ST5 *n* = 9, and ST8 *n* = 3), or penicillin and erythromycin (ST30, *n* = 3), penicillin, erythromycin and sulfadimethoxine (ST5 *n* = 4), and penicillin, tetracycline, and sulfadimethoxine (ST7 *n* = 1). None of the cow source isolates were resistant to more than one antibiotic class (Additional File 1). One BTM isolate (ST5) was resistant to penicillin and sulphadimethoxine (Additional File 1). The remaining isolates were sensitive to all antibiotics tested. The presence of *blaZ* was detected in all 24 beta-lactam-resistant isolates. Antimicrobial susceptibility was tested by MIC and disc diffusion methods and the results were 100% concordant.

**Fig. 3:**
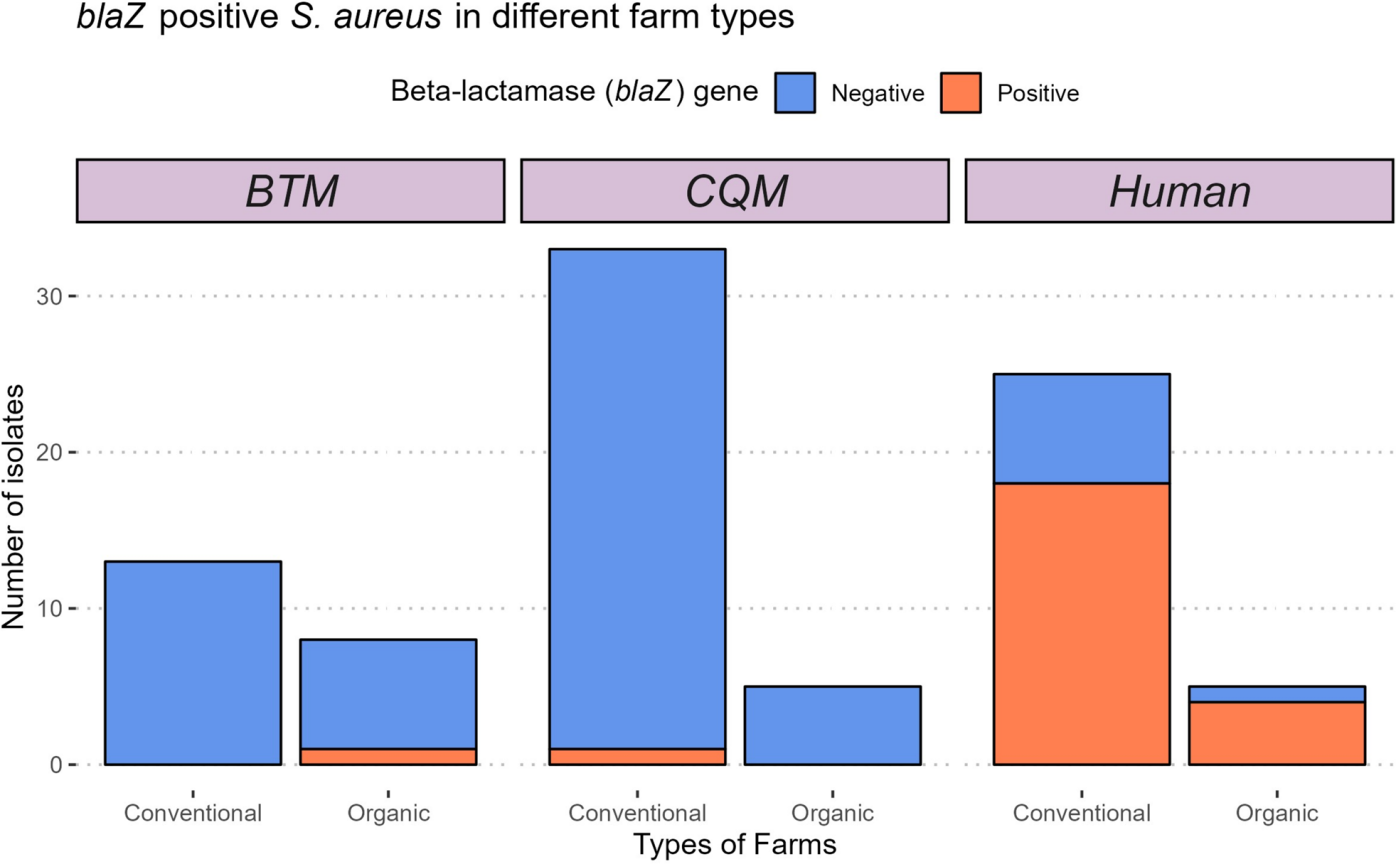
Frequency of the beta-lactam resistance among *S. aureus* isolates by farm type and isolate source. *S. aureus* frequency stratified by beta-lactam gene amplicon PCR results, the farm type (Organic Vs. Conventional) of the isolate origin, and the three different sources of isolates on the farms. (BTM: Bulk tank milk, CQM: Cow quarter milk, Human: hand and nasal swabs)

**Fig. 4:**
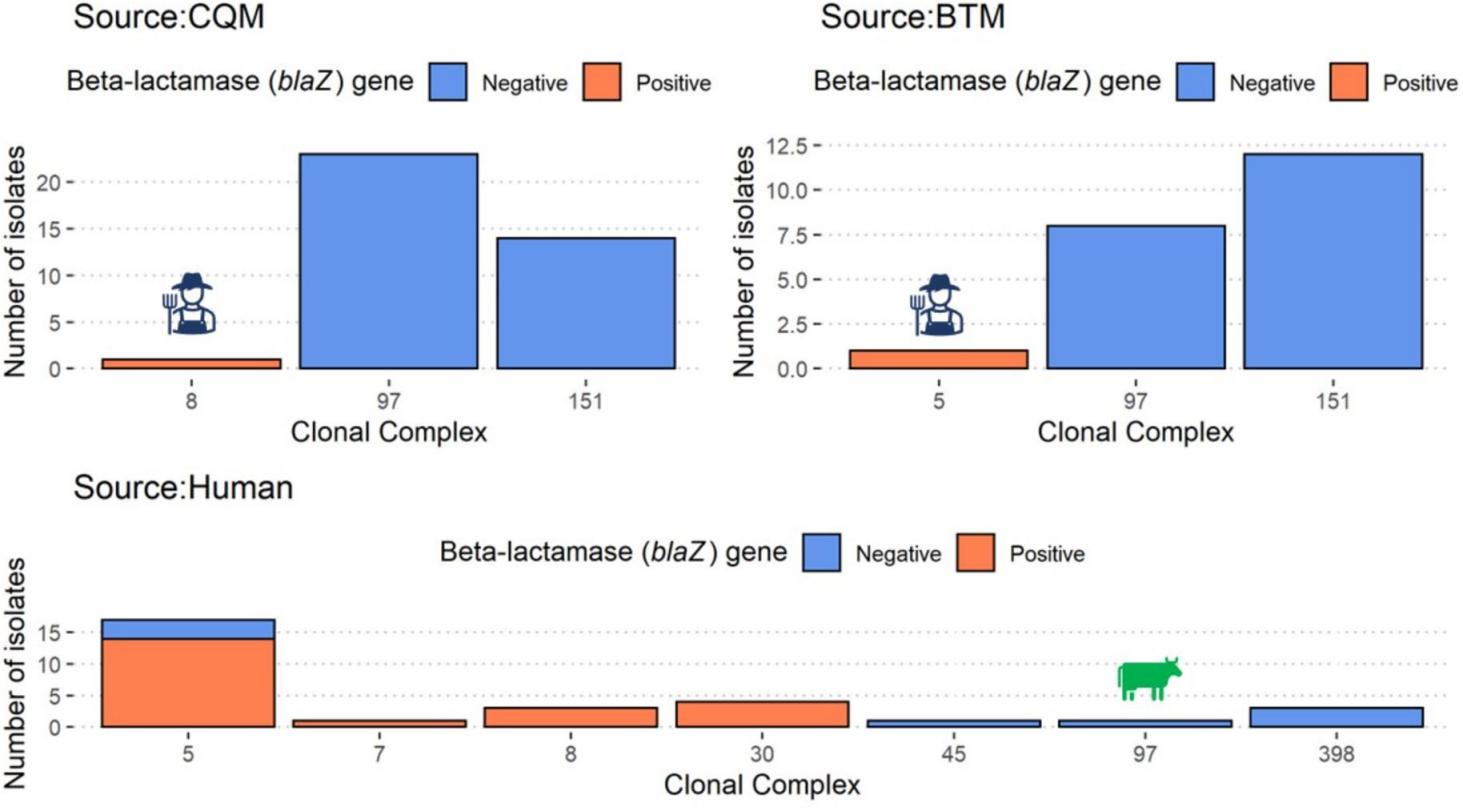
Frequency of the beta-lactam resistance among *S. aureus* isolates by isolate source and clonal complex. Frequency of the beta-lactam resistance gene presence (*blaZ* positive) among *S. aureus* isolates from 3 different sources stratified by MLST clonal complex. (BTM: Bulk tank milk, CQM: Cow quarter milk, Human: hand and nasal swabs). The human and cow icons identify potential spillover isolates, which are defined as host-adapted isolates associated with one host species and isolated from a different host species

No isolates were *mecA-*positive or phenotypically resistant to cefoxitin. Beta-lactam-resistant *S. aureus* isolates were found on 47.36% (9/19) of the farms. In the tests of association, ST and CC (Clonal Complex) and farm type were not predictors of *blaZ* status. However, the source was a significant predictor. On organic farms, 1/13 isolates obtained from CQM and BTM were *blaZ*-positive, while 4/5 isolates from human swab samples were *blaZ-*positive. On conventional farms, 1/46 isolates obtained from CQM and BTM were *blaZ*-positive, while 18/25 isolates from human swab samples were *blaZ*-positive (Fig. 3). The *blaZ*-positive isolates were cultured from 2/3 humans working on organic farms and 8/11 humans working on conventional farms (one isolate collected from a human on a conventional farm was lost in storage before AST). Pearson’s chi-squared and Likelihood Ratio G tests showed strong evidence of an association between *S. aureus blaZ* PCR status and the source from which the bacterium was isolated, with *blaZ* more prevalent among human isolates (73%) compared to CQM isolates (2.63%) or BTM isolates (4.76%). The beta-lactam-resistant isolates belonged to 6 STs and 4 CCs (CC5, CC7, CC8, and CC30). All CC151 isolates obtained from CQM and BTM were beta-lactam susceptible and *blaZ* negative (Fig. 4). Tetracycline- and erythromycin-resistant *S. aureus* strains were isolated from only one and three farms, respectively. All three ST398 isolates were erythromycin-resistant, and one was lincomycin-resistant. One ST151 isolated from BTM was pirlimycin- and lincomycin-resistant (i.e., lincosamide-resistant).

## Discussion

### Frequency of *S. aureus* on dairy farms

The frequency of *S. aureus*-positive BTM samples in our study is consistent with earlier studies from Minnesota and Ohio, which reported 63% and 69% prevalence, respectively [27, 28]. Because cows with *S. aureus* mastitis shed into milk, BTM samples are considered helpful for estimating the herd status of *S. aureus* and evaluating milk quality and udder health [29, 30].

The cow-level frequency of *S. aureus* in our study was 5.9% (35/589), similar to earlier studies that reported a prevalence of 4.8% in conventional farms and 6.8% in organic farms [31]. The frequency estimate of our research is likely biased upwards as nine participating herds submitted farmer-collected quarter milk samples from suspect mastitis cases. Among this subset of cows, the *S. aureus* prevalence was 10%. In contrast, we completed whole herd sampling of all lactating cows from eight participating herds, where the cow level *S. aureus* prevalence for this subset of herds was 4%.

The frequency of *S. aureus* carriers among farmers was 36.6%, which is consistent with a study showing a prevalence of 38% among animal caretakers [5]. While these studies provide numerical estimates of colonization of farm works that are greater than estimates of 25–30% often reported for the general population [8], further study is needed to determine if farmstead cheese producers are colonized at greater frequencies than the general population. Other studies have indicated that some professions, such as hospital workers [32], veterinarians [33], and cheese plant workers [34], have higher frequencies of *S. aureus* colonization, suggesting additional studies of dairy farm workers are justified. Further, in future studies, US organic farms may represent a comparator farm population where livestock antibiotic use is severely limited.

Overall, we observed higher frequency of isolation of *S. aureus* from the BTM samples compared to humans or individual cattle. This finding is not surprising given that the bulk tank milk is a composite sample of multiple lactating cows in a herd so a single cow in a herd shedding *S. aureus* into their milk can result in a positive BTM culture [19, 30]. Further, BTM may be *S. aureus* negative despite having *S. aureus* infected cows in a herd due to intermittent milk shedding of cows with *S. aureus* intramammary infections [30]. When the individual cows or humans were clustered at the farm level, then the frequency of isolating *S. aureus* from each of the three sources (humans, cows, or BTM) did not differ on the participating Vermont farms.

### Multi Locus Sequence Typing (MLST) profiles

Strain typing of *S. aureus* isolates from the three sources, humans, cow quarter milk and comingled bulk tank milk, demonstrated an association between strain type and source, consistent with prior studies suggesting *S. aureus* strains are host-adapted [3–5, 23]. In our study, with rare exceptions, we isolated different strains from humans compared to cows and milk on the farms (i.e., the strain types were associated with the source of the isolates). Further, the strains isolated from bulk tank milk represented the strains isolated from individual cow quarter milk samples within a farm. These results suggest that there may be barriers to spillover and host switching of *S. aureus* host-adapted strains between humans and cattle, such as the acquisition of virulence factors allowing adaptation to the new host species [16, 20, 35], (further discussed below under potential spillover events).

Like other studies, we observed multiple *S. aureus* strains from dairy cattle and milk in a defined geographic region [36, 37]. In Ireland, 18 STs were identified from mastitis-associated isolates on 26 farms, with 84% in CC97 and CC151 [36]. In Pennsylvania, 16 STs were identified from isolates of BTM and mastitis cases on 77 herds, with 94% in CC97 and CC151 [37]. Similarly, we found eight STs from BTM and CQM samples on 14 farms, with 96% in CC97 and CC151. In our research and a previous study from Pennsylvania, the STs found in BTM appeared to be the same STs causing intramammary infections. Four STs belonging to CC97 were isolated from 63.15% of the farms (*n* = 12), demonstrating the preponderance of this *S. aureus* lineage in cheesemaking Vermont dairy farms. The dominance of CC97 among bovine isolates has also been observed globally [35]. Four of the five novel strains found in this study belonged to bovine-associated CC97 and CC151, further demonstrating the dominance of these bovine-associated lineages in our sample population of dairy cattle.

Previous studies have sampled farm workers on dairy farms, identifying ST398, ST45, ST8, ST30, ST25, ST5, ST72, and ST121 [3, 4]. Our study is novel in that we collected isolates from workers on conventional and organic dairy farms and identified similar STs from farm workers on both farm types. CC5 was the most dominant human-associated CC, comprising ST5 and ST5957. We also isolated ST398 from human nose and hand swabs from a single farm. ST398 was first isolated from pig farmers in Europe [38] and has been isolated from humans in studies conducted in the USA [39, 40].

### Antimicrobial Sensitivity

In our study, most isolates were sulfadimethoxine-resistant, which is consistent with other studies [41, 42]. This high level of resistance could be attributed to using sulfadimethoxine to treat pneumonia and foot infections in veterinary medicine in the USA [43]. We also found isolates resistant to beta-lactams, tetracycline, and erythromycin, which is in line with other studies that have reported similar resistance patterns for *S. aureus* on dairy farms [4, 37, 42]. We isolated erythromycin-resistant MSSA ST398 from farm workers, which is consistent with a previous study [4].

We identified no methicillin-resistant isolates based on phenotypic testing of cefoxitin and oxacillin and *mecA*-PCR amplicon screening. We did not screen for *mecC* DNA sequences in this study, as phenotypic screening revealed no cefoxitin resistance. While *mecA*-gene-positive isolates may display phenotypic susceptibility to oxacillin, phenotypic susceptibility to cefoxitin is sufficient to screen for the presence of *mecA*- and *mecC-*MRSA [45].

The beta-lactam-resistant isolates in our study were more likely to be collected from farm workers on both organic and conventional farms. These isolates belonged to CC5, CC8, CC7, and CC30, defined as human host-adapted lineages, consistent with previous studies conducted on dairy farms [4, 17, 44]. In comparison, beta-lactam resistance was infrequent among cow and BTM-sourced isolates belonging to cattle host-adapted strains CC97 and CC151.

We observed two challenges with testing associations between beta-lactam resistance, source, and STs or CCs. First, the contingency table, tabulating the isolate source and CCs, contained several null values (e.g., no CC7, CC30, CC45, or CC398 isolated from cows or no CC151 isolated from humans). This is presumably due to the host-specific nature of CCs, coupled with the fact that we defined the source of isolates according to the host (human, cow, or BTM). Because CCs are host-associated, we explored the association between CC and the source of isolates, which indicated a moderate to strong association (Cramer’s V = 0.67). This result created a second challenge of multicollinearity between predictors of interest in our modeling approach. Here, we took the simple approach to resolving this issue by considering the source as a predictor in a univariate model. Our observation that beta-lactam resistance was more frequently identified in human host-adapted clonal complexes deserves additional study. A limitation of our current study is the small sample size relative to the number of CCs, perhaps explaining the lack of association between CCs and beta-lactam resistance. The presence of beta-lactam-resistant isolates among humans on US organic dairy farms offers an opportunity to study antibiotic-resistant pathogen transfer between humans and cattle without antibiotic use in livestock. In the United States, antibiotic use is not allowed on organic dairy farms, and cows requiring antibiotic treatment are removed from the farm [9], suggesting that organic dairy farms have reduced selective pressure for developing or spreading resistant bacteria. Therefore, the study of organic farm systems may be used to quantify the potential for humans to be a source of resistant pathogens in agriculture.

Taken together, these results provide additional evidence that dairy farms serve as reservoirs for antibiotic-resistant *S. aureus* strains that can spread between cattle, humans, and the environment. Integrated surveillance platforms and mitigation strategies guided by One Health principles are essential to control the selection and dissemination of antibiotic resistance across interconnected animal and human populations. Implementing antimicrobial stewardship on dairy farms may reduce the pressure for selection and maintenance of antibiotic-resistant *S. aureus* at the human-cattle interface, and Ruegg has outlined an approach to implementing antimicrobial stewardship on dairy farms [46]. Additional research is needed to understand the potential One Health benefits of antimicrobial stewardship on dairy farms making farmstead cheeses, and the potential role that humans may play disseminating resistant elements to livestock.

### Potential Spillover Events

Yebra et al., distinguished transient spillover (jump between host species without onward transmission to other individuals in the new host species) from host switching (jump between species with onward transmission in the new host population) [35]. Our study found instances of possible spillover, defined as the isolation of a host-associated ST in an alternative host species. For example, an isolate belonging to ST352 (CC97) was cultured from a human-hand swab, and human-associated CC5 was present in a BTM sample. An isolate belonging to CC8 was cultured from a CQM sample. CC8 has been isolated from cows with mastitis and is speculated to have recently jumped from humans to cattle in other geographic regions [3, 6, 47, 48]. Other studies have also documented the recent transfer of CC5 and CC97 between humans and cows [3, 4, 6, 47]. The pathways to zoonotic spillovers and host switching have been reviewed [49, 50]. In the dairy farm environment, many steps in the path to spillover are met. Humans and dairy cattle are in frequent close contact, with multiple direct daily contacts, especially during milking. Further, during cheese making, humans directly contact milk and cheese. Both humans and cattle can be colonized or infected with and shed *S. aureus*. *S. aureus* strains may have or can acquire the capacity to overcome host-specific barriers to infection [47, 48, 51]. Contacts between humans and cattle are especially close during hand-milking or milking preparation when udders are stripped by hand. Wearing and frequently changing disposable gloves during milking are recommended milking hygiene practices to reduce contagious mastitis pathogen transmission between cows, which might also contribute to reduced frequency of spillovers or host switching [52]. A limitation of our study is we only conducted single farm visits, which makes it impossible to confirm whether the isolates were transiently present or permanently colonized in their alternative hosts. Future studies should include longitudinal designs to identify transient spillovers or host switching in real time and identify best practices to prevent spillover events. We speculate that some current best practices for miking time hygiene such as pre- and post-milking teat disinfection and milkers wearing disposable gloves while milking cows are key practices to limiting between host transmission. All farms in this study reported implementation of these practices and some of the farms had implemented active *S. aureus* mastitis surveillance and control practices in their herds. Our work provides additional support to the concept that strain typing of *S. aureus* can help identify potential sources of infection or contamination in humans and cattle on dairy farms [53].

*S. aureus* spillover has implications for antibiotic resistance spread [50]. Antibiotic use in farm systems contributes to the emergence of antibiotic-resistant pathogens of human and animal health concern. While our study did not identify MRSA strains, livestock-associated MRSA CC398 causes human infections with evidence for bidirectional exchange [54, 55]. In our research, antimicrobial resistance was infrequent among cattle-associated *S. aureus* strains. Both cases of possible spillover of human-associated *S. aureus* strains isolated from milk samples were beta-lactam resistant, suggesting humans as a potential reservoir of antibiotic-resistant *S. aureus* in dairy production systems, consistent with prior conclusions of Schmidt et al. [6, 17]. To the best of our knowledge, no previous studies of AMR staphylococci isolated from cattle and humans on the same farms could infer the direction of transmission [3, 56–58]. It is a logical hypothesis that dairy farm milker hygiene and biosecurity practices are critical for mitigating spillover risk.

## Conclusions

Our study provides insights into the prevalence and clonal diversity of *S. aureus* strains among hand skin and nasal swabs of dairy workers and milk of cows on cheesemaking farms in Vermont. We found that humans were more likely to carry beta-lactam-resistant *S. aureus* than cows. On organic farms, only human-adapted *blaZ*-positive STs were isolated from BTM. Moreover, we identified potential spillover events of *S. aureus* sequence types between host species. These findings support the importance of the One Health Initiative for continued monitoring of *S. aureus* at the human-animal interface.

## Methods

The Strengthening the Reporting of Observational Studies in Epidemiology–Veterinary Extension (STROBE-Vet) statement guidelines were followed in the reporting of this study [59].

### Study design, setting, and participants

In this observational study, 19 Vermont dairy farms that produce farmstead cheese or milk for artisan cheese production were selected through a non-probability convenience sample design. No formal sample size calculation was performed before the start of this study, although a priori, our goal was to sample more than 15 herds and 40 farm workers (2 to 3 people per herd). Eligible farms were identified from a publicly available member list of a cheese producer organization and a contact list of producers who previously participated in research projects with the University of Vermont. Certified organic and conventional dairy herds from Vermont were eligible to participate. There were no restrictions based on other demographics (e.g., herd size, breed, age of farm, or farmer characteristics). During the study period, the total number of dairy farms in Vermont ranged from approximately 850 in 2015 to 725 in 2018, and the number of on-farm dairy processors ranged from 71 (2015) to 63 (2018). An estimated 50 farms made farmstead cheese, and 25 farms provided milk to off-farm artisan cheese producers during the study period. Thirty-seven herds were contacted with information on the study objectives. Nineteen herds, approximately 25% of Vermont farms producing milk for farmstead or artisan cheese, volunteered to participate, and samples were collected in February and March between 2013 and 2015 (5 herds) and from June to August 2018 (14 herds). Each farm was visited once for sample collection. Informed consent was obtained from all participants, and the study was approved by the University of Vermont's Committee on Human Subjects Research (protocol CHRMS 14–512) and Institutional Animal Care and Use Committee (protocol 13–033).

### Sample Collection

The samples included human-nasal and -hand swabs, quarter milk (CQM) from lactating cows, and composite bulk tank milk (BTM). The farm employees self-swabbed both anterior nares with a single sterile nylon-flocked swab (FLOQSwabs #502CS01, Copan Diagnostics Inc., or PurFlock Ultra #25–3506-U, Puritan Medical Products) according to the procedures described by Gamblin et al. [60]. Laboratory personnel collected hand swab samples from employees and nasal swabs from farm dogs. All swab samples were refrigerated for up to 48 h or stored at -20 °C for up to 90 days before processing.

Individual CQM samples were collected a) by farmers from selected cows with known or suspected mastitis or previous intramammary infections (*n* = 9 herds), or b) by laboratory personal sampling all lactating cows in the herd (*n* = 8 herds). The sampling was performed using guidelines from the University of Minnesota Laboratory for Udder Health Milk Sample Collection Guide [61]. For farmer-collected samples, the samples were stored frozen on the farm for up to 2 weeks before being transported to our research laboratory. For quarter milk samples collected by laboratory personnel, the samples were held on ice during transport back to the laboratory, refrigerated overnight, and cultured within 24 h of collection. Two farms did not contribute CQM samples.

Farm visits were scheduled when the bulk tanks contained milk from at least two consecutive milkings. Laboratory personnel collected 250 ml herd-level bulk tank milk samples after 5 min of agitation of the bulk tank and stored them in a sterile single-use vial (Sterlin™ Dippa™ #192, Thermo Scientific). All specimens were transported on ice to the laboratory and stored at -20 ºC up to 90 days before processing.

### Bacterial culture

The samples collected from humans, dogs, and BTM were grown on non-selective tryptic soy agar with 5% sheep blood (TSAWB) as well as three selective media: mannitol salt agar (MSA), chromogenic *S. aureus* agar (CHRSA), and chromogenic MRSA agar (CHRMRSA). Individual CQM samples were cultured on TSAWB according to established guidelines [62]. All plates were incubated at 37 °C for 24 h, except for TSAWB plates, which were incubated for 48 h. For swab samples, the swabs in transport solution were first vortexed, aseptically removed from the vial using flame-sterilized forceps, and then directly swabbed onto TSAWB plates. Serial dilution of remaining swab suspension (undiluted, tenfold, and 100-fold in sterile water) was prepared, and 100 µl of each solution was spread onto TSAWB (undiluted, 1:10, 1:100), MSA (undiluted, 1:10, 1:100), CHRSA (undiluted), and CHRMRSA (undiluted) using L-shaped stick. Additionally, 500 µl of the swab inoculated suspension was inoculated into 4.5 ml sterile Mueller–Hinton broth containing 6.5% sodium chloride for enrichment. After enrichment at 37 °C for 24 h, serial dilutions of 1:1000 and 1:10,000 were prepared, and 100 µl was spread on TSAWB, CHRSA, and CHRMRSA. For bulk tank milk samples, the methods for inoculation on different plates with and without dilution was as described above for swab samples.

### Presumptive Isolation and Identification of *Staphylococcus aureus*

Individual colonies resembling staphylococci based on their growth characteristics (i.e., colony morphology, color, size, hemolysis pattern, mannitol fermentation on MSA, and pigmentation on CHRSA and CHRMRSA) were picked and inoculated onto new TSAWB plates to isolate for purity. Between 2 and 6 representative colonies of presumptive *S. aureus* isolates were selected from each primary culture plate. Presumptive identification criteria for staphylococci included: round colonies 2–3 mm in diameter, opaque greyish white, white, pale yellow, or golden yellow colonies generally hemolytic on TSAWB; clear to white or yellow colonies that ferment mannitol on MSA; mauve to pink colonies on CHRSA and CHRMRSA. After incubation at 37 °C for 48 h, the hemolytic pattern on TSAWB was observed, followed by gram staining, catalase, and coagulase tests of each presumptive isolate. Presumptive *S. aureus* was gram-positive, catalase and coagulase tests positive, and cocci with complete and/or partial hemolysis on blood agar plates. Occasional non-hemolytic, gram-positive, catalase-positive, and coagulase-positive isolates were identified and stored for subsequent species identification by PCR. Presumptive isolates that were gram-positive, catalase-positive, and coagulase-negative cocci (e.g., non-aureus staphylococci) and gram-positive pleomorphic rods (e.g., *Corynebacteria* spp.) were also occasional selected from the primary culture plates and stored. Presumptive isolates were frozen at -20 or -80 °C in sterile tryptic soy broth with 15% glycerol until further processing. Isolates were revived from frozen stock by plating 10 µl on TSAWB, incubating for 48 h, and passing in culture to a new plate to confirm purity before subsequent identification.

### DNA extraction and Multiplex PCR

The genomic DNA of the isolates was extracted using the Qiagen DNeasy Blood and Tissue Kit. Multiplex PCR, using three pairs of primers, was performed to confirm presumptive isolates to be *S. aureus* with the presence of thermonuclease (*nuc*) gene and to identify *blaZ* and *mecA* gene carried by those confirmed isolates (see Additional file 1). Positive DNA template controls (*S. aureus* ATCC 25923, ATCC 29213, and ATCC 33591) and negative template controls (nuclease-free PCR water) were included for each amplification. The presence of PCR products of the approximate size was determined by visualizing SYBR Safe-stained 1.5% agarose gels after electrophoresis.

### Multilocus Sequence Typing (MLST)

For MLST analysis, genomic DNA from all *nuc*-positive isolates was subjected to PCR using primers for seven housekeeping genes specific to *S. aureus* [21]. The amplified DNA was cleaned using ExoSAP-IT PCR clean-up (Affymetrix) and then subjected to Sanger sequencing at the University of Vermont Genomics Core Facility. The reverse and forward chromatograms were aligned and screened for quality using *Geneious Prime®* software (version 2022.1.1, Biomatters Ltd.). Amplicons with poor-quality sequences or alignments with mismatches were re-sequenced. Consensus sequences were queried against the *S. aureus* MLST database (https://pubmlst.org/) to determine allele and sequence type matches. Novel alleles or allelic profiles were submitted to the MLST database curator for new allele and ST number assignment. All identified isolates were submitted to the database.

### Antimicrobial susceptibility testing (AST)

Antimicrobial sensitivity testing was performed using agar disc diffusion (DD) and broth microdilution assays, following CLSI guidelines [63] with 20 antibiotics (see Additional file 1). Broth microdilution assays were performed using a commercially available 96-well plate (Sensititre Mastitis MIC plates, CMV1AMAF, Trek Diagnostic Systems), while agar disc diffusion assays were performed using commercially available discs. *S. aureus* ATCC 25923 and ATCC 29213 were used as quality control strains for disc diffusion and broth microdilution assays, respectively. The disc diffusion and Sensititre plate results were interpreted according to CLSI guidelines [42, 63]. Because CLSI does not provide breakpoints for *S. aureus* from mastitis cases for many of the antibiotics tested, no categorical breakpoint definitions were applied for those antibiotics, and the observed quantitative results (MIC or zone diameter) were reported for all antibiotics tested. We defined multi-drug resistant isolates as those resistant to two or more antibiotic classes.

### Data management and Statistical analysis

In this study, we defined isolates as bacterial colonies selected from primary culture plates and subcultured on secondary plates showing homogenous morphology. Sequence types (STs) were defined as isolates with a common MLST allelic profile, and clonal complex (CC) was defined as a group of closely related STs with five or more similar alleles [21, 53].

Isolates of the same MLST type, with the same AMR profile, isolated from the same individual source on the same farm were defined as duplicates and excluded from statistical analysis to avoid over-representation of strain diversity and provide unbiased antimicrobial resistance prevalence estimates [64–66]. The number of duplicate isolates within ST, cow, and farm was recorded (see Additional file 1). For example, if we collected four isolates from an individual quarter of a cow on one farm, and these isolates had the same ST and AMR profile, then one representative isolate was used in the analysis. For each isolate, nominal categorical variables included the source of isolate (human, CQM, or BTM), originating farm type (conventional or organic), CC, and *blaZ* PCR status (negative or positive).

*Geneious Prime®* software (version 2022.1.1, Biomatters Ltd.) was used to create pseudogenes by concatenating the allele sequences of the housekeeping genes. A phylogenetic tree of pseudogenes was created using MEGA (Molecular Evolutionary Genetics Analysis) version 6.0 [67]. The minimum spanning tree was constructed using PHYLOViz [68]. Statistical tests of association were done using R (R version 4.2.2, The R Foundation for Statistical Computing Platform). In a forward stepwise regression approach, we explored the association between the presence of *blaZ* (as a proxy for antimicrobial resistance of human and animal health concern) and each categorical independent variable (source, farm type, and strain type or clonal complex) in univariate models. Variables with *P* < 0.20 were brought forward to a multivariable regression model. In the final models, interactions or associations were considered significant with a *P* < 0.05. The degree of association between the predictor variables clonal complex and source was tested using Cramer’s V statistic for categorical variables.

### Supplementary Information

**Supplementary Material 1.**

## Data Availability

All data generated or analyzed during this study are included in this published article and its supplementary information files (Additional File 1). The isolate data are available at the PubMLST (https://pubmlst.org/bigsdb?db=pubmlst_saureus_isolates) *S. aureus* public databases for molecular typing.
